# Supplementary material for: Neural Correlates of Learning Pure Tones or Natural Sounds in the Auditory Cortex
Source: Front Neural Circuits. 2020 Jan 28;13:82. doi: 10.3389/fncir.2019.00082 (PMC6997498; doi:10.3389/fncir.2019.00082)
Supplement: Supplementary file 2 [file Presentation_1.pdf]

CAPTIONS FOR SUPPLEMENTARY MOVIE

Supplementary Movie 1-

Representative example of a mouse while training in the Educage. **Right**: Top view of the Educage chamber, which is located on the left side. The Educage is connected to the home cage via a plastic tube. The video shows a mouse entering the Educage for three consecutive trials and then returning to the home cage. The sounds played are the ‘Go’ or ‘No-Go’ sounds. **Left**: Top view of the licking port showing the nose pokes and licking of the mouse. The first two trials are ‘Hit’ trials where the mouse receives a water reward. The third trial (a ‘Correct reject’ trial) shows that the mouse withholds licking in response to the non-target tone.

SUPPLEMENTARY FIGURES

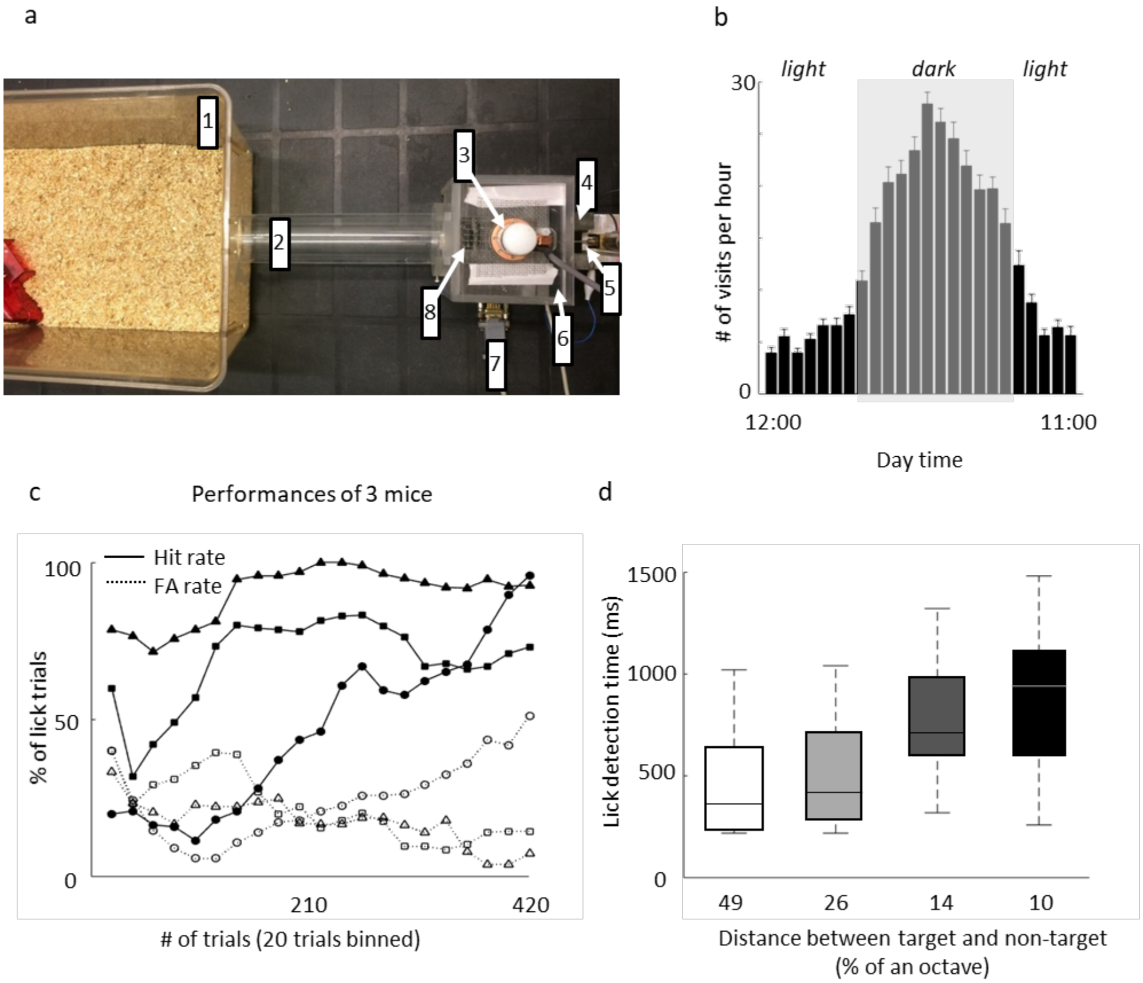

Supplementary figure 1-

a. Photograph of the Educage platform (top view). 1. Home cage; 2. Connecting tunnel; 3. Auditory speaker; 4. IR diodes monitoring the behavioral port; 5. Water spout and licko-meter; 6. RFID antenna; 7. Connection to D/A converter; 8. Electrically conductive grid floor (for lickometer circuit). **b.** Histogram of daily hourly distribution of voluntary training. Gray area represents dark hours. N=39 mice (mean ± s.e.m). **c.** Representative examples of the performance of three mice (the same mice shown in ‘Figure 1B’) during the first stage of discrimination. The graph shows % of lick responses to the target tone (‘Hit rate’-solid line) and to the non-target tone (‘FA rate’- dashed line). **d.** Population averages of detection times for the different discrimination levels. N=12 mice (mean ± s.e.m). Shades denote the level of difficulty (from 49% to 10% octave apart).

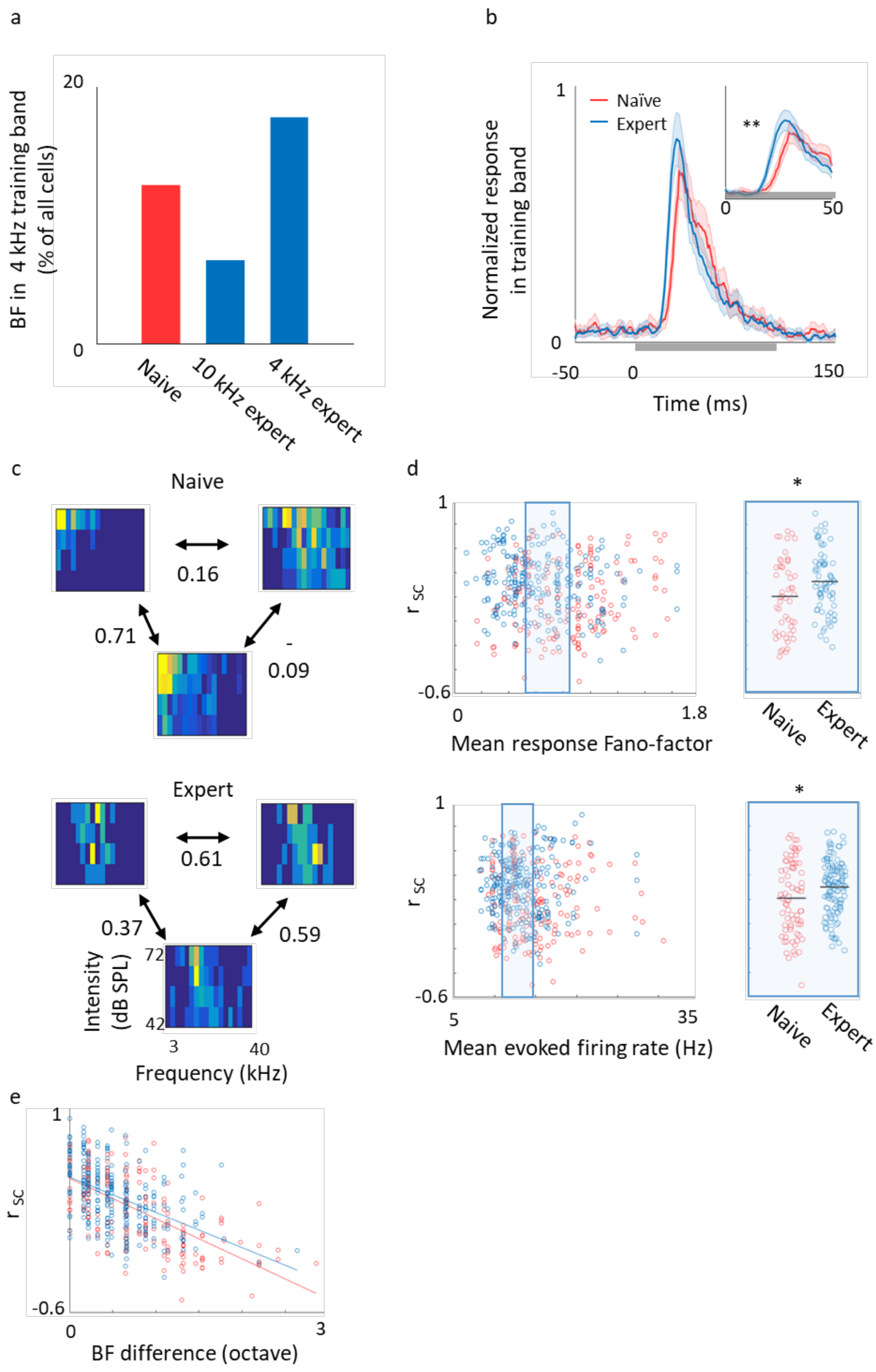

Supplementary Figure 2

Supplementary figure 2 -

a. The fraction of neurons with BF that resides within a 4kHz frequency band in three different experimental groups. Naïve, (n=22 mice) - red; mice trained on a 10kHz (n=20 mice), and 4kHz (n=4 mice) as the target tone - blue. **b.** Population average of normalized PSTHs from naïve (red) and expert mice trained (blue; mean ± s.e.m). The PSTHs shown were collapsed for all intensities and for all frequencies inside the training band, binned at 1 ms time bins. Inset: zoom-in on the first 50 ms from stimulus onset. **c.** Representative examples of frequency response areas (FRA's) and pairwise signal correlations (r_{SC}) of neighboring neurons from the same local circuits in naïve (top) and expert (bottom) mice. **d.** Left, Scatter plot of pairwise r_{sc} and pair average of evoked firing rate or response Fano-factor for naive (red circles) and expert (blue circles). The range common to the naïve and expert (0.5 SD bellow and above the median) is indicated as blue rectangle. Right, All pairwise r_{sc} values from the common range show consistent differences between groups, independent of these parameters (Mann-Whitney U-test: * $p<0.05$). **e.** Scatter plot of pairwise r_{sc} and pair best frequency difference showing strong dependency (Naïve: slope=-0.3; p=2E^{-28}; R^2= 0.42; Expert: slope=-0.27; p=5.8E^{-17}; R^2=0.22).

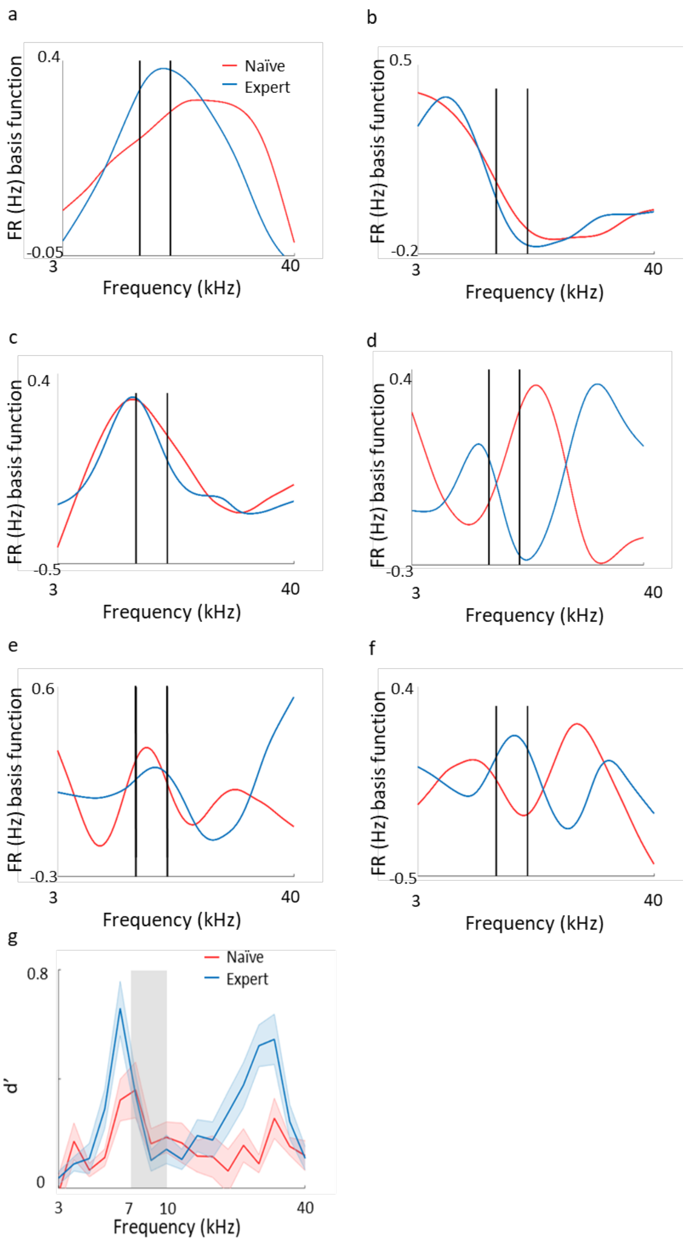

Supplementary figure 3 -

a-f. 6 basis functions coming out of the SVD analysis for naïve (red) and expert (blue) neurons. **g.** Neuronal d' computed from the raw firing rate from naïve (red) and expert (blue) animals.

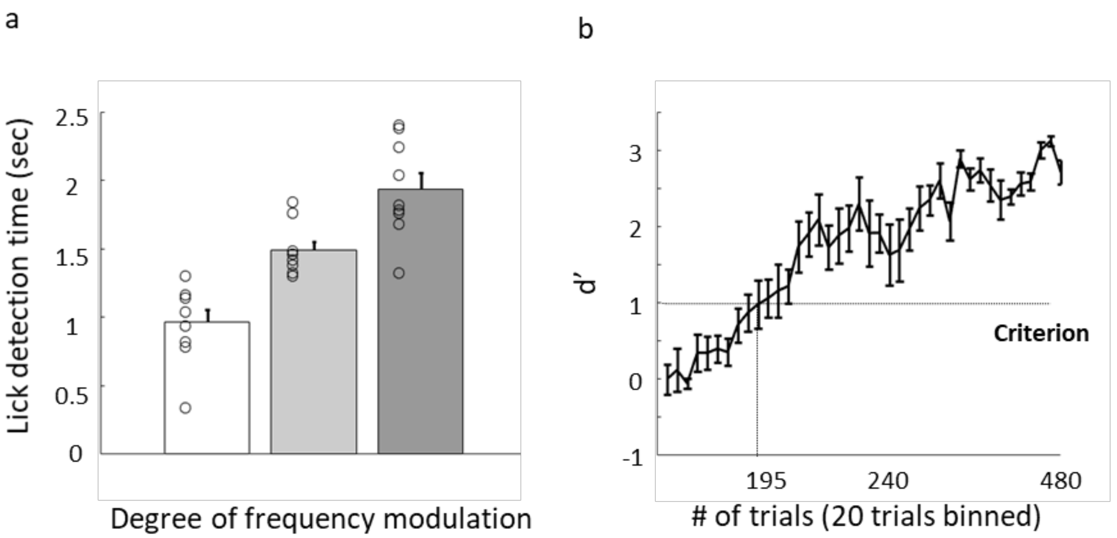

Supplementary figure 4 -

a. Population average of detection times for the different discrimination stages. N=9 mice (mean ± s.e.m). Shades denote the level of difficulty. **b.** d' values (mean± s.e.m) for the first stage of discrimination (WC vs Reverse). The learning criterion is represented as dashed line (d'=1).

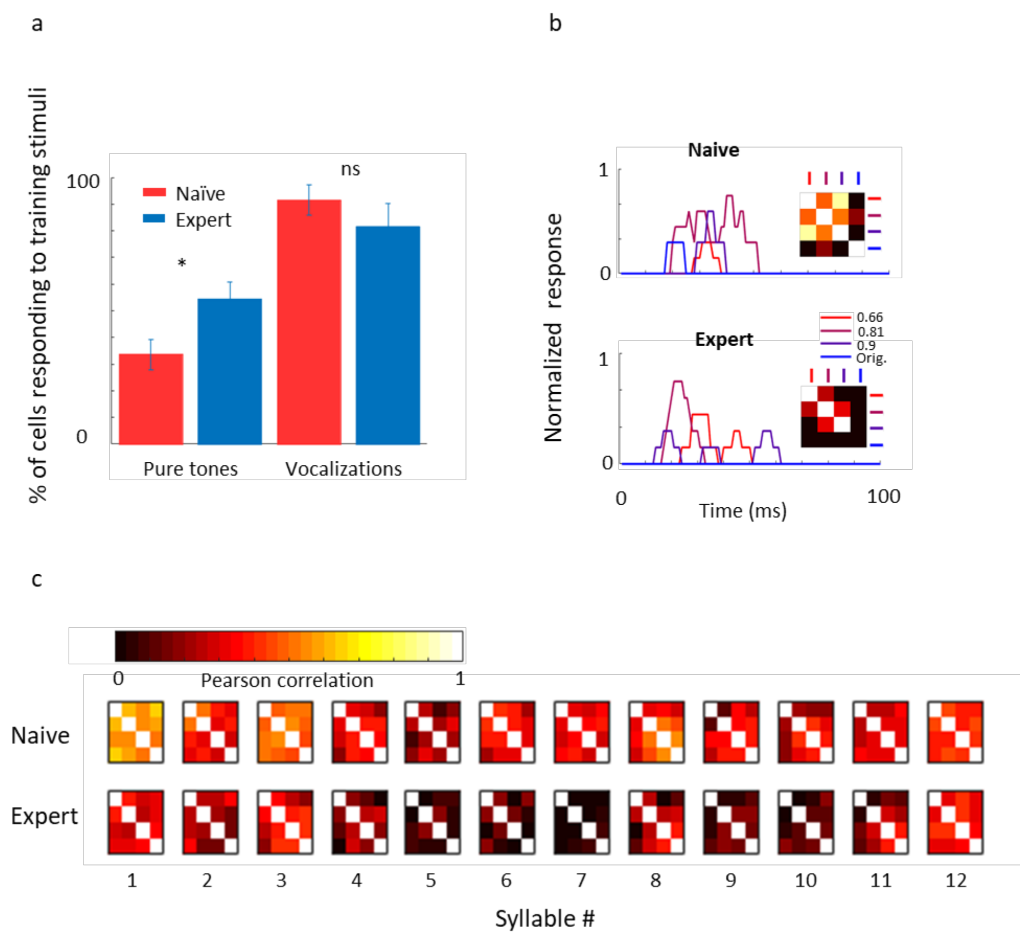

Supplementary Figure 5

Supplementary Figure 5 -

a. Fraction of cells with significant response to frequencies within the training band (left) and to the original WC in naïve (red) and expert (blue) mice (mean ± s.e.m). Fraction of cells responding to the trained vocalization remained constant (Mann-Whitney U-test: p=0.09) **b.** PSTHs for two representative naïve (top) and expert (bottom) neurons responding to different modulations of syllable #4. **Inset**. Correlation matrices for these PSTHs. **c.** Correlation matrices for different syllables individually. Each pixel indicates the average Pearson correlation value from two different calls calculated from the evoked spike rate from all neurons in response to one syllable. Neurons from expert mice have lower correlation between responses to different modulated calls, and specifically in syllables 5-7.

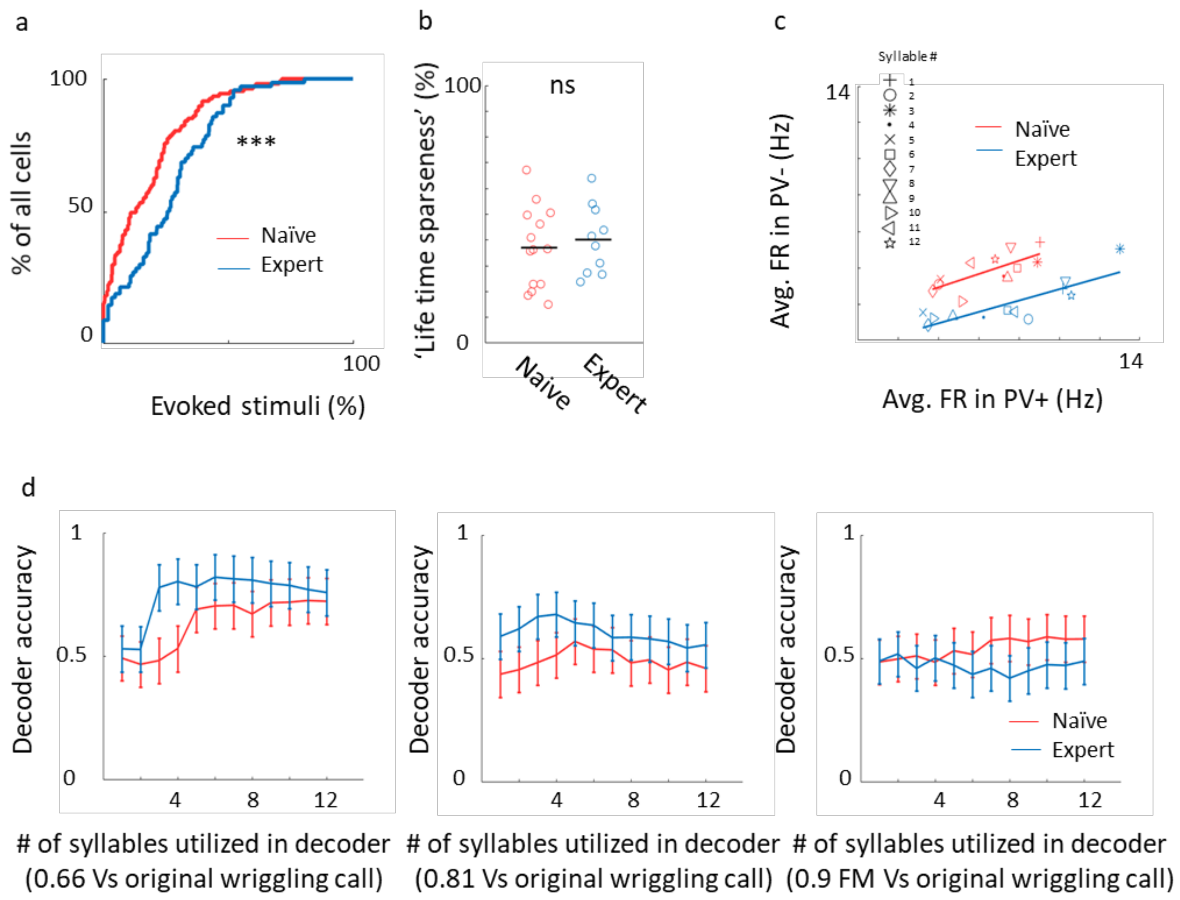

Supplementary Figure 6-

a. Cumulative distribution of response selectivity of PV^+ neurons in naïve (red) and expert (blue) mice. Response selectivity was determined as the % of all frequency-intensity combinations that evoked significant response. PV^+ neurons in expert were less selective (Kolmogorov Smirnov test; p=0.0015). **b.** Life time sparseness for all cells from the middle range of the firing rate distribution show no significant difference between naïve (red circles) and expert (blue circles) groups (Mann-Whitney U-test; p=0.46). **c.** Scatter plot showing linear dependency of average syllable evoked firing rate of PV^- and PV^+ neurons in naïve (red; R^2=0.5; slope=0.37; p=0.008) and expert (blue red; R^2=0.7; slope=0.31; p<0.001) mice. Each marker corresponds to the average response for one syllable in the sentence**. d.** Classification performance of a Support Vector Machine (SVM) decoder based on PV^+ responses.

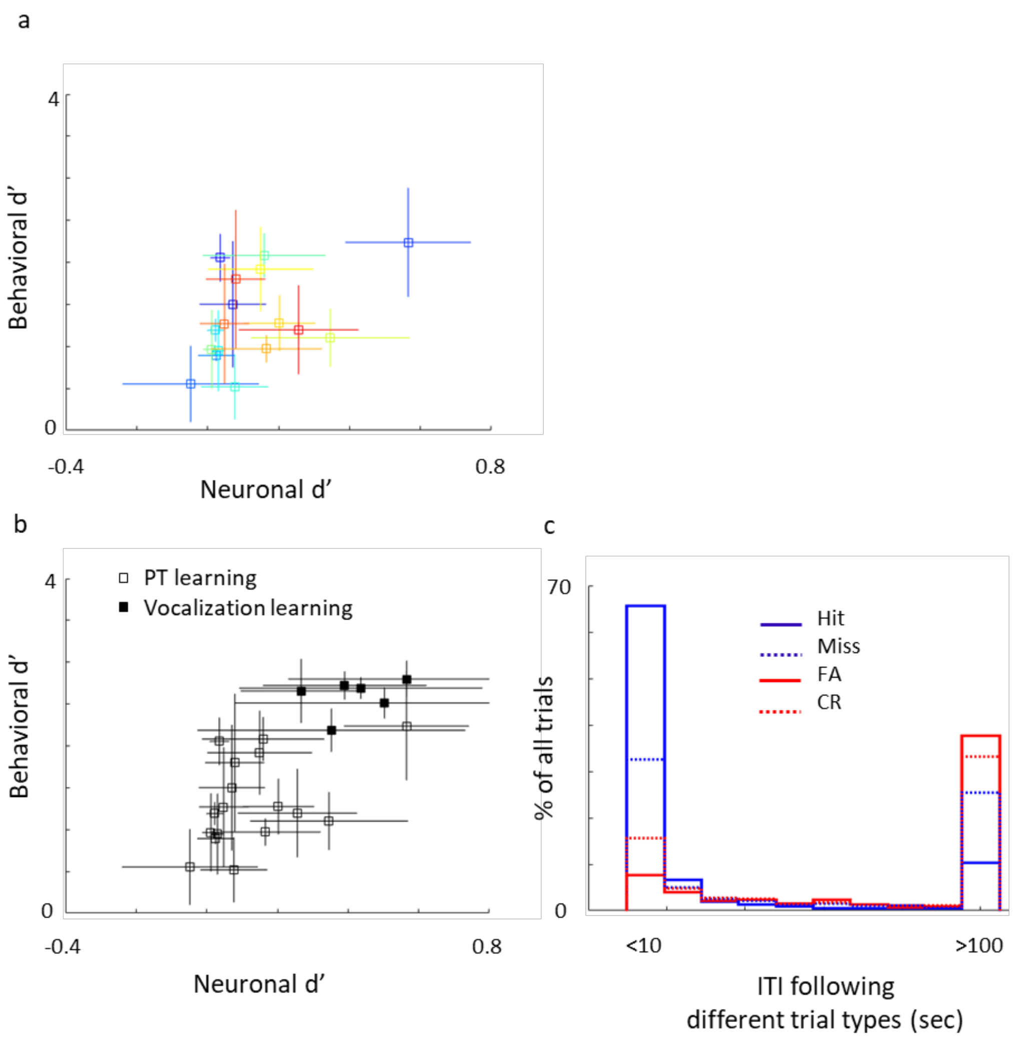

Supplementary Figure 7-

a. Correlation between behavioral d' and neuronal d' of individual mice (mean±std; r^2=0.15, p=0.07). Colors denote the same mice and neurons as in Supplementary Figure 2f. **b.** Same as in A but also for mice trained on vocalization task. **c.** Histograms of inter-trial intervals (ITI) following 'Hit' (solid blue), 'Miss' (dashed blue), 'FA' (solid red) and 'CR' (dashed red) trials.
